# Supplementary material for: Representative Test Material for Validation of Density Separation as Part of Microplastic Quantification in Drinking Water
Source: Polymers (Basel). 2025 Feb 18;17(4):526. doi: 10.3390/polym17040526 (PMC11858966; doi:10.3390/polym17040526)
Supplement: Supplementary file 1 [file polymers-17-00526-s001.zip › polymers-3385169-supplementary.pdf]

Supplementary Material

Figure S1: Bruker T-Star TXRF detailed analysis and analysis method configuration

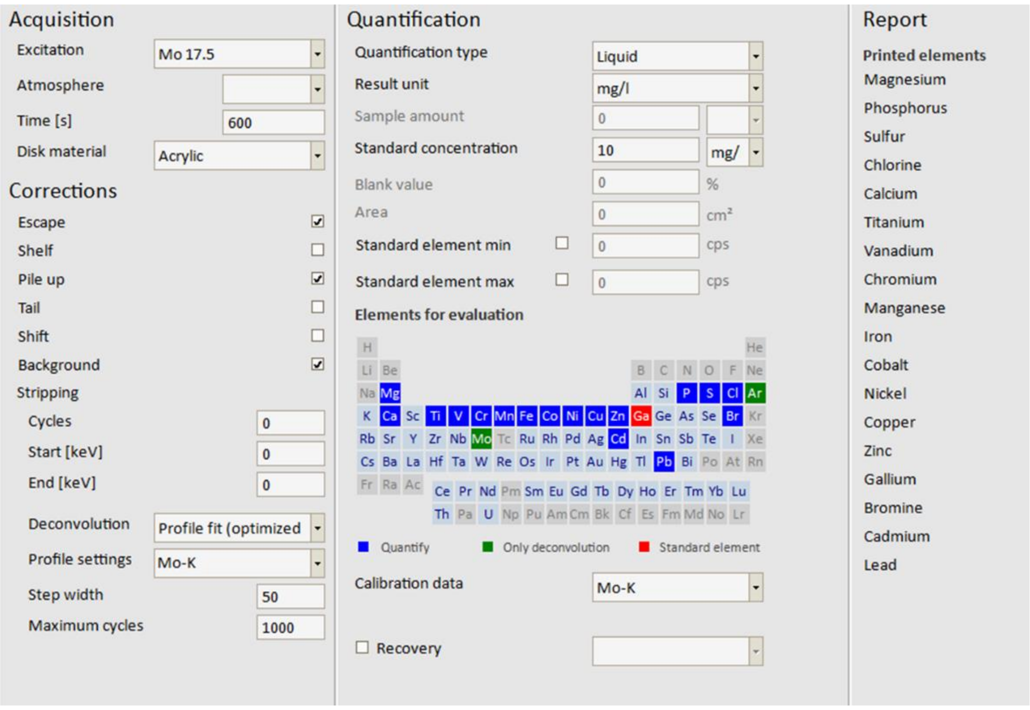

Table S1: Agilent 7700x detailed instrument and method configuration for multi-elemental semi-quantitative analysis

Parameter	Value
**Introduction system**	Micromist nebulizer 0.4 mL min^{-1}<br>Scott double-pass spray chamber set at 2°C<br>Quartz torch with 2.5 mm id injector<br>Platinum sampling and skimmer cones<br>Uptake tubing Tygon 1.02 mm id (white/white)<br>Drain tubing PharMed 1.52 mm id (yellow/blue)
**Plasma power**	1550 W
**Sampling depth**	8 mm
**Plasma gas flow (Ar)**	15 L min^{-1}
**Auxiliary gas flow (Ar)**	0.9 L min^{-1}
**Nebulization (carrier) gas flow (Ar)**	1.0 mL min^{-1}
**He collision gas flow**	4 mL min^{-1}
**Analytes**	39 elements* in semi-quantitative mode (calibration based on 25 elements from a multi-elemental standard solution and extrapolated for 14 other elements absent from the solution)
**Wash between injections (Rinse 1)**	5% HNO_3
**Probe rinse port**	H_2O

*Li, Be, K, Ca, Sc, Cr, Zn, Mo, Ru, Cd, Sn, Eu, Se, Rb, Y, Cl, Si, Co, Ni, Cu, Na, Al, B, Mn, Fe, Mg, Au, Ti, As, Pb, Zr, V, Pd, Ba, Ag, Sr, In, Rh, Bi

Figure S2: Raman and FT-IR spectra of JRCNM70508a and control PVC with the most prominent vibration modes assigned to wavenumber values

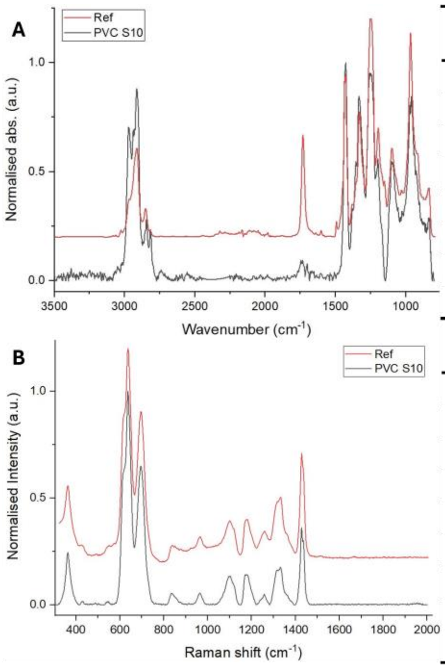

Wavenumber (cm⁻¹)	Correlation
837	C-Cl stretching
964	C-H out of plane deformation
1097	C-C stretching
1251	CH-Cl out of plane angular deformation
1331	CH_2 deformation group
1431	C-H aliphatic vending
2817	CH_2 symmetric stretching
2970	CH_2 symmetric stretching

Raman Shift (cm⁻¹)	Correlation
637	C-Cl stretching in $CH_2 = CHCl$
695	C-Cl symmetric stretching in $CH_2 = CHCl$
966	C-Cl asymmetric stretching in $CH_2 = CHCl$
1101	C-O stretching
1180	CH_2 twist
1260	C-H rocking
1332	CH bend out of pase IR + CH_2 deformation
1428	CH_2 bend in pase IR
2910	C-H stretching

The parameters used in the density separation experiment were optimised previously carrying out only the separation step to diverse of analytes, obtaining solid recoveries (82.4±11.1 % for 51 µm PS particles and 92-9 ± 3.2 % with sand), as well as running theoretical calculations based on the Stokes Law. The next table (Table S3) presents some of them:

Table S3: Recoveries obtained for different analytes submitted to the centrifugal density separation.

Analyte	Recovery (%)	SD	Conditions
PS 51 µm	82.4	11.1	1620 rpm 20 min 20°C
PS 51 µm + 10 mg sand	92.9	3.2	1620 rpm 4 min 20°C
Nylon Fiber 25 x 900 µm	81.6	5.5	1620 rpm 10 min 20°C
